# Disruption of zinc transporter ZnT3 transcriptional activity and synaptic vesicular zinc in the brain of Huntington’s disease transgenic mouse

**DOI:** 10.1186/s13578-020-00459-3

**Published:** 2020-09-11

**Authors:** Li Niu, Li Li, Shiming Yang, Weixi Wang, Cuifang Ye, He Li

**Affiliations:** 1 Department of Histology and Embryology, Tongji Medical College, Huazhong University of Science and Technology, 13 # Hangkong Road, Wuhan, 430030 People’s Republic of China; 2 School of Biomedical Sciences, LKS Faculty of Medicine, the University of Hong Kong, Hong Kong S.A.R., People’s Republic of China; 3 Hubei Key Laboratory of Embryonic Stem Cell Research, Hubei University of Medicine, Shiyan, 442000 People’s Republic of China

**Keywords:** Synaptic vesicular zinc, Zinc transporter 3, Sp1, Transcriptional inhibition, Synaptic dysfunction, Huntingtin, Huntington’s disease

## Abstract

**Background:**

Huntington’s disease (HD) is a neurodegenerative disease that involves a complex combination of psychiatric, cognitive and motor impairments. Synaptic dysfunction has been implicated in HD pathogenesis. However, the mechanisms have not been clearly delineated. Synaptic vesicular zinc is closely linked to modulating synaptic transmission and maintaining cognitive ability. It is significant to assess zinc homeostasis for further revealing the pathogenesis of synaptic dysfunction and cognitive impairment in HD.

**Results:**

Histochemical staining by autometallography indicated that synaptic vesicular zinc was decreased in the hippocampus, cortex and striatum of N171-82Q HD transgenic mice. Analyses by immunohistochemistry, Western blot and RT-PCR found that the expression of zinc transporter 3 (ZnT3) required for transport of zinc into synaptic vesicles was obviously reduced in these three brain regions of the HD mice aged from 14 to 20 weeks  and  BHK  cells  expressing  mutant  huntingtin. Significantly, dual-luciferase reporter gene and chromatin immunoprecipitation assays demonstrated that transcription factor Sp1 could activate ZnT3 transcription via its binding to the GC boxes in ZnT3 promoter. Moreover, mutant huntingtin was found to inhibit the binding of Sp1 to the promoter of ZnT3 and down-regulate ZnT3 expression, and the decline in ZnT3 expression could be ameliorated through overexpression of Sp1.

**Conclusions:**

This is first study to reveal a significant loss of synaptic vesicular zinc and a decline in ZnT3 transcriptional activity in the HD transgenic mice. Our work sheds a novel mechanistic insight into pathogenesis of HD that mutant huntingtin down-regulates expression of ZnT3 through inhibiting binding of Sp1 to the promoter of ZnT3 gene, causing disruption of synaptic vesicular zinc homeostasis. Disrupted vesicular zinc ultimately leads to early synaptic dysfunction and cognitive deficits in HD. It is also suggested that maintaining normal synaptic vesicular zinc concentration is a potential therapeutic strategy for HD.

## Background

Huntington’s disease (HD) is an autosomal dominant neurodegenerative disease caused by the pathological expansion of CAG repeats (> 36) in the first exon of the HD gene encoding huntingtin (Htt) [1–7]. Neuropathologically, the neuronal loss occurs primarily in the striatum and the cortex in the early stages of HD. However, other brain regions, such as the hippocampus, hypothalamus, brainstem, and spinal cord, are also affected in advanced stages [1–5]. HD symptoms occur usually in mid-life, comprising movement, cognitive and psychiatric impairments inexorably progressing to death within two decades [6]. Psychiatric deteriorations and cognitive deficits precede the onset of motor disorder [5, 6], and even the onset of both psychiatric and motor symptoms often occur years prior to detectable neuronal loss [6].

Synaptic dysfunction greatly contributes to HD pathogenesis [7–9]. Neurotransmitter release significantly alters in the transgenic HD mouse models [10, 11]. Neuronal and synaptic dysfunction precedes cell death by many years in the HD patients [7, 8] and animal models [9]. Furthermore, some pharmacological interventions focus on targeting early synaptic disturbances, which has been proven to restore synaptic function [12–14] and delay progression to neurodegeneration in HD transgenic mice [15, 16]. Therefore, disturbed synaptic function accounts for the early symptoms of HD and triggers neuronal death in later stages of the disease [17, 18]. It is vital to investigate the mechanism of synaptic damage in HD disease.

The divalent cation zinc in the brain contributes to efficient synaptic transmission. Approximately, 85% of total brain zinc is tightly bound to metalloproteins. 10–15% of total brain zinc is highly localized in synaptic vesicles of excitatory glutamatergic neurons [19, 20]. This pool of zinc, the ionic zinc, is either free or chelatable. It can be detected with simple histochemical method such as neo-Timm’s sulfide-silver method [21, 22], and is thus often called histochemically reactive zinc. Neurons containing histochemically detectable zinc are present in many regions of the brain, including the neocortex, striatum, hippocampus, amygdale and olfactory bulb [23]. Vesicular zinc is closely linked to modulating synaptic transmission. It serves as a singal factor to play an important role in modifying glutamatergic neurons [24]. Zinc releases from glutamatergic neuron terminals, which may protect neurons from excitotoxicity of glutamate to attenuate the excess amount of presynaptic glutamate release [25]. Zinc deficiency affects neurogenesis and trigger neuronal apoptosis. Therefore, this can result in learning and memory deficits [25].

More improtantly, the homeostasis of zinc in the brain is tightly regulated. The zinc transporters (ZnTs) mainly function to efflux zinc out of cytoplasm or into intracellular organelles [26]. Among them, zinc transporter 3 (ZnT3), the primary vesicular zinc transporter, is located on the membrane of synaptic vesicles to transport zinc ions into presynaptic vesicles from the cytosol [26]. The concentration of vesicular zinc depends on the abundance of ZnT3 [26, 27]. Targeted deletion of ZnT3 gene eliminates zinc from synaptic vesicle, which leads to age-dependent deficits in learning and memory ability [27] and neurodegeneration [27, 28]. Consequently, ZnT3-dependent zinc homeostasis in synaptic vesicles takes an important role in maintaining synaptic function.

An imbalance of vesicular zinc homeostasis is associated with the pathogenesis of multiple neurodegenerative diseases, including Parkinson’s disease (PD) [29], Alzheimer’s disease (AD) [30] and amyotrophic lateral sclerosis (ALS) [31]. These diseases have in common with HD features and mechanisms that the misdolded proteins cause neuronal death at the late onset of the disorder. Altered homeostasis of essential elements such as iron, chromium and selenium has been observed in the HD patients [32, 33] and mice model [34]. Specially, increased level of zinc is detected in the blood of HD patients, indicating that mutant Htt (mHtt) might impair zinc homeostasis [32]. Nevertheless, it remains unclear whether mHtt affect zinc homeostasis in the brain. Here, we report that mHtt reduces ZnT3 expression by inhibiting the binding of Sp1 to ZnT3 promoter and down-regulates vesicular zinc level in the brain of N171-82Q HD transgenic (TG) mice. It is therefore suggested that disruption of vesicular zinc homeostasis may ultimately contribute to synaptic dysfunction and neurodegeneration in HD.

## Results

### N171-82Q HD transgenic mice display significant loss of total zinc and vesicular zinc in the brain tissue

The flame atomic absorption spectrometry (FAAS) and autometallography (AMG) were respectively applied to explore the effect of mHtt on total and vesicular zinc level in the brain tissue of TG mice. Comparison of the 20-week-old TG mice with age-matched wild type (WT) mice showed that total zinc level was extremely low in the cortex, striatum and hippocampus examined in the HD mice, compared to controls (Additional file 1: Figure S1).

**Fig. 1:**
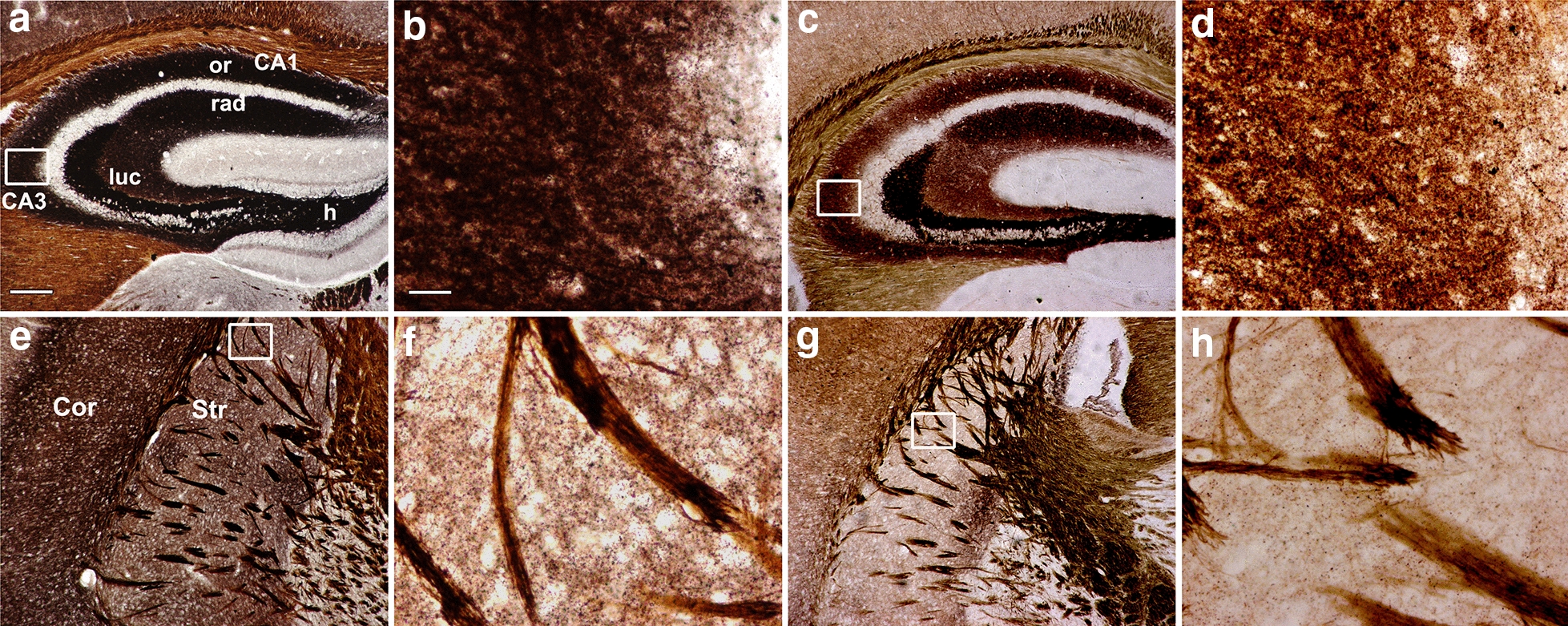
Light microscopic images of vesicular zinc in TG mice brain. AMG stain is abundant in hippocampus and striatum of the 20-week-old WT mice (**a, b**, hippocampus; **e, f**, striatum), but very week in age-matched TG mice brain (**c, d** , hippocampus; **g, h**, striatum). **b, d, f** and **h** are the magnified images indicated by boxed areas in **a, c, e** and **g**, respectively. *h* hilus, *luc* s *lucidum*; or: s oriens; *rad* s radiatum, *Cor* cortex, *Str* striatum. Scale bars: 500 μm in **a, c, e** and **g**; 125 μm in **b, d, f** and **h**

AMG results under light microscope showed that histochemically reactive zinc was significantly decreased in all three brain regions examined in the 20-week-old TG mice, compared to controls (Fig. 1). In the wild type (WT) mice, intense AMG staining was seen in the CA1, CA2 and CA3 region of hippocampus (Fig. 1a, b), cortex (Fig. 1e) and striatum (Fig. 1e, f). However, the HD mice presented faint zinc staining in the corresponding brain regions (Fig. 1c, d, g, h).

**Fig. 2:**
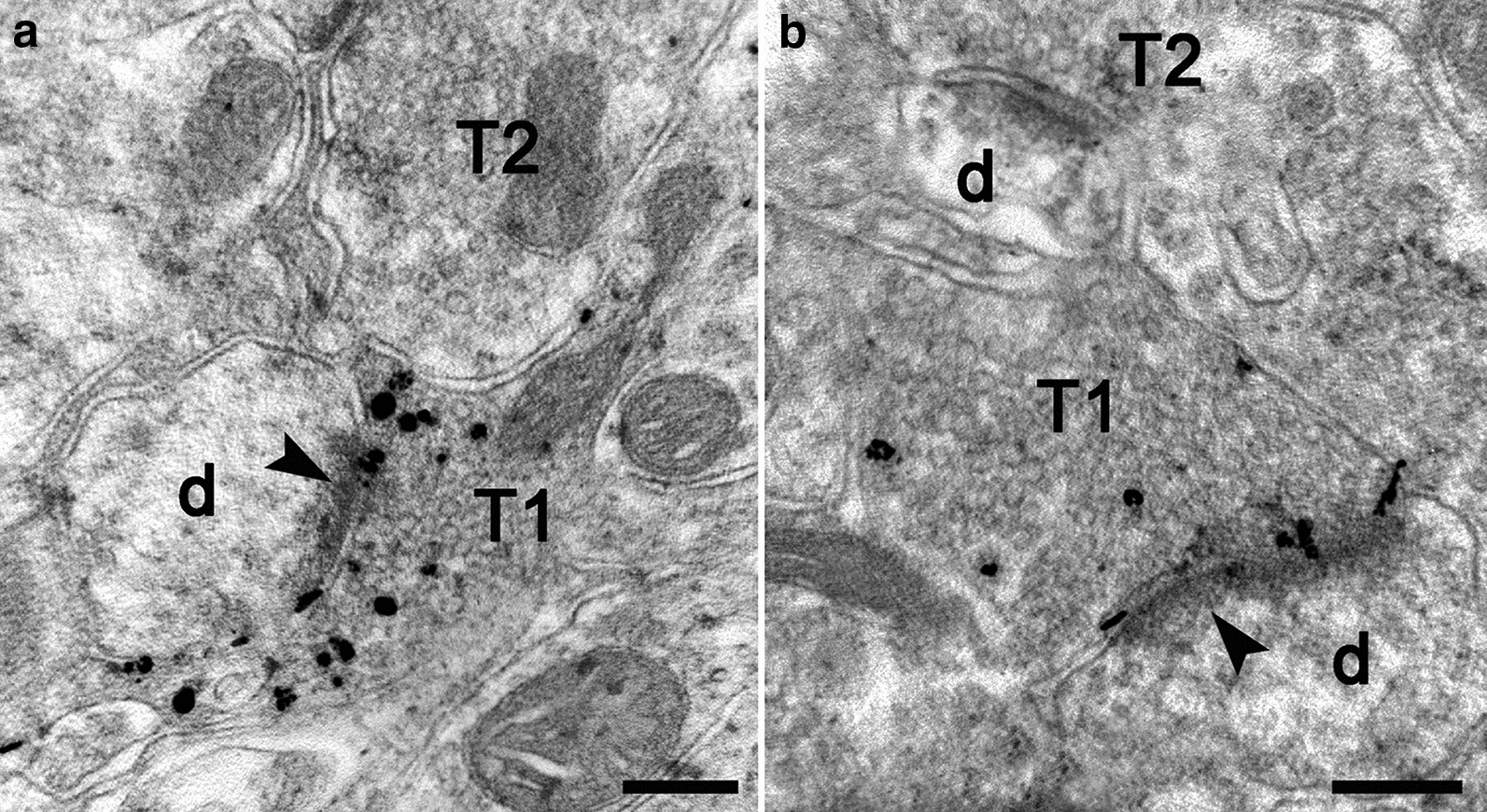
Electron microscopic images of vesicular zinc in the striatum of TG mice. Zinc density in synaptic vesicles dramatically decreases in the striatum of the 20-week-old TG mice (**b**) compared to age-matched WT mice (**a**). Arrowheads show postsynaptic membrane. *T1* axonal terminal containing AMG positive granules; *T2* axonal terminal without AMG positive granules, *d* dendrite. Scale bars: 0.2 μm

Under electron microscope, the AMG reactive zinc granules were present within synaptic vesicles in the striatum of the WT mice (Fig. 2a), whereas the number of positive vesicles was rarely seen in the striatum of the HD mice (Fig. 2b). These collective findings indicates that zinc is unbalanced in the HD brain and suggests a role of zinc in HD pathogenesis.

### ZnT3 expression is decreased in N171-82Q HD transgenic mice and BHK cells expressing mutant huntingtin

**Fig. 3:**
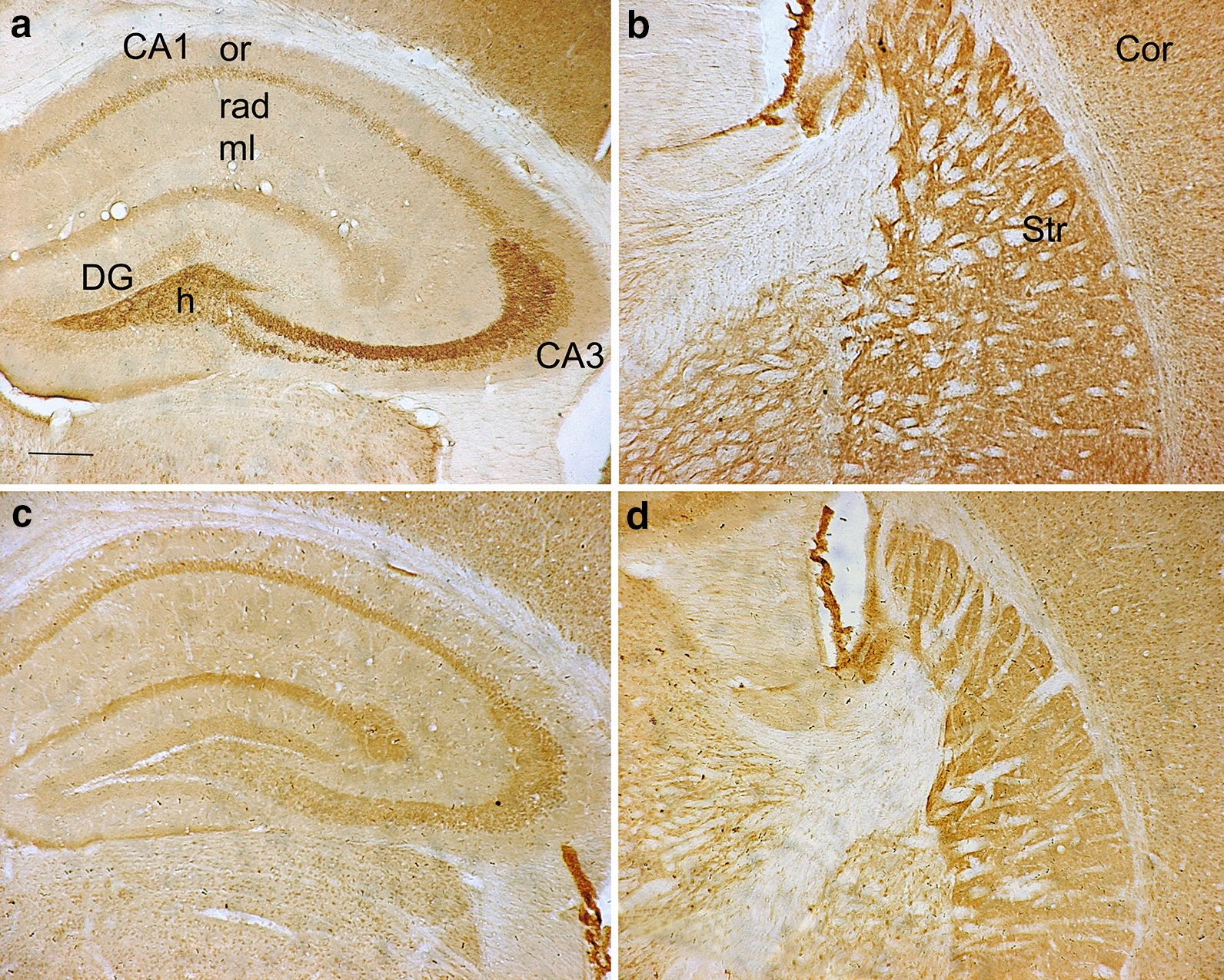
Immunohistochemistry determination of ZnT3 expression in TG mice brain. The 20-week-old TG mice (**c** hippocampus, **d** striatum) display weak ZnT3-immunoreactivities in the brain, compared to age-matched WT mice (**a** hippocampus, **b** striatum). *DG* dentate gyrus, *ml* molecular layer. Scale bar: 500 μm

ZnT3 is required for transport of zinc into synaptic vesicles and the amount of ZnT3 on synaptic vesicle membranes regulates concentration of vesicular zinc [27]. To identify whether the decrease of vesicular zinc in HD brain is due to impairment of ZnT3 expression, we examined ZnT3 level in TG mice brains and BHK cells expressing N-terminal mHtt containing 160 glutamine repeats (160Q cells). Light microscopic immunohistochemistry revealed that ZnT3 immunoreactivities were intense in the hippocampus, striatum and cortex of the 20-week-old WT mice (Fig. 3a, b). In contrast, only weak ZnT3-immunoreactivities were seen in the same brain regions of age-matched TG mice (Fig. 3c, d).

**Fig. 4:**
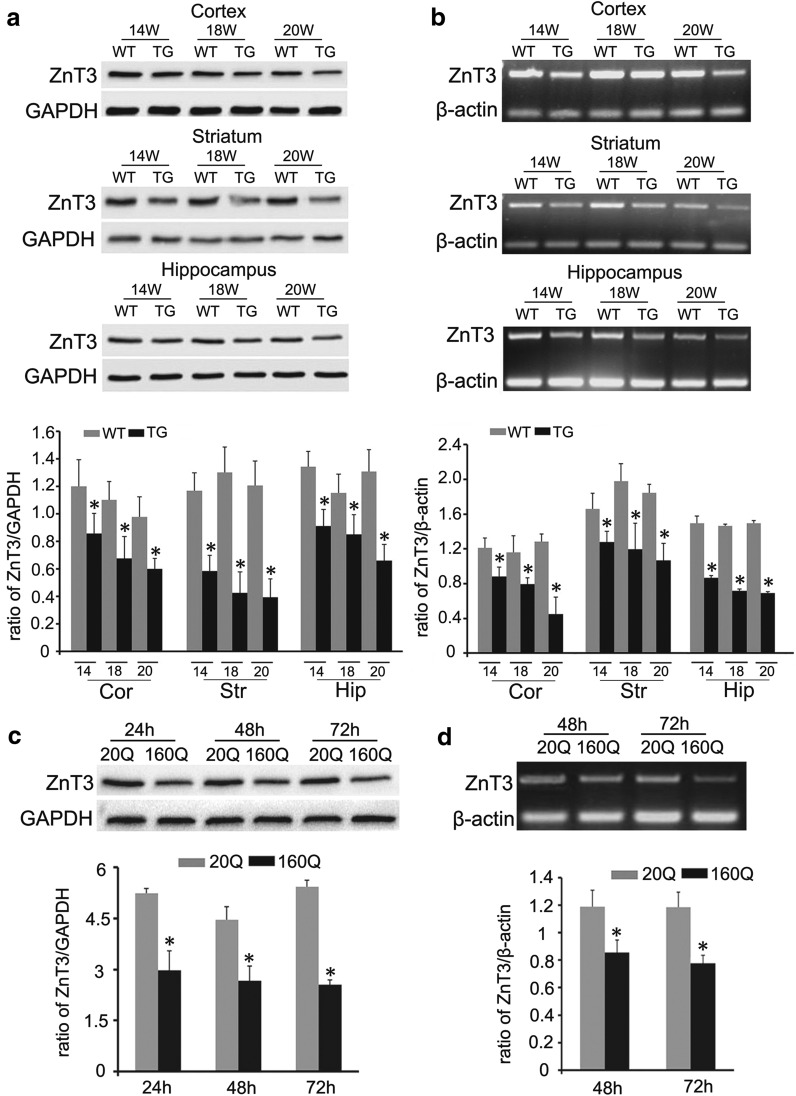
Mutant huntingtin impairs ZnT3 protein and mRNA expression. **a, b** The expression of ZnT3 is decreased in cortex, striatum and hippocampus of TG mice aged from 14 to 20 weeks, compared to age-matched WT mice (**a** Western blot analysis, **b** RT-PCR analysis). *Hip* hippocampus. n = 4. *p < 0.05 compared to WT mice. **c, d** The expression of ZnT3 is decreased in 160Q BHK cells at different times after transfection, compared to 20Q BHK cells (**c** Western blot analysis, **d** RT-PCR analysis). n = 4. *p < 0.05 compared to 20Q BHK cells

Further, Western blot and RT-PCR analyses found that the protein and mRNA levels of ZnT3 in the cortex, hippocampus and striatum were markedly reduced in 14-, 18-, and 20-week-old TG mice in comparison with age-matched WT controls (Fig. 4a, b). In the 160Q cells, decreased protein and mRNA levels of ZnT3 were also detected by Western blot and RT-PCR (Fig. 4c, d). Therefore, it is reasonable to conclude that mHtt causes the loss of vesicular zinc by down-regulating ZnT3 expression.

### Sp1 positively regulates ZnT3 expression through activating ZnT3 promotor

**Fig. 5:**
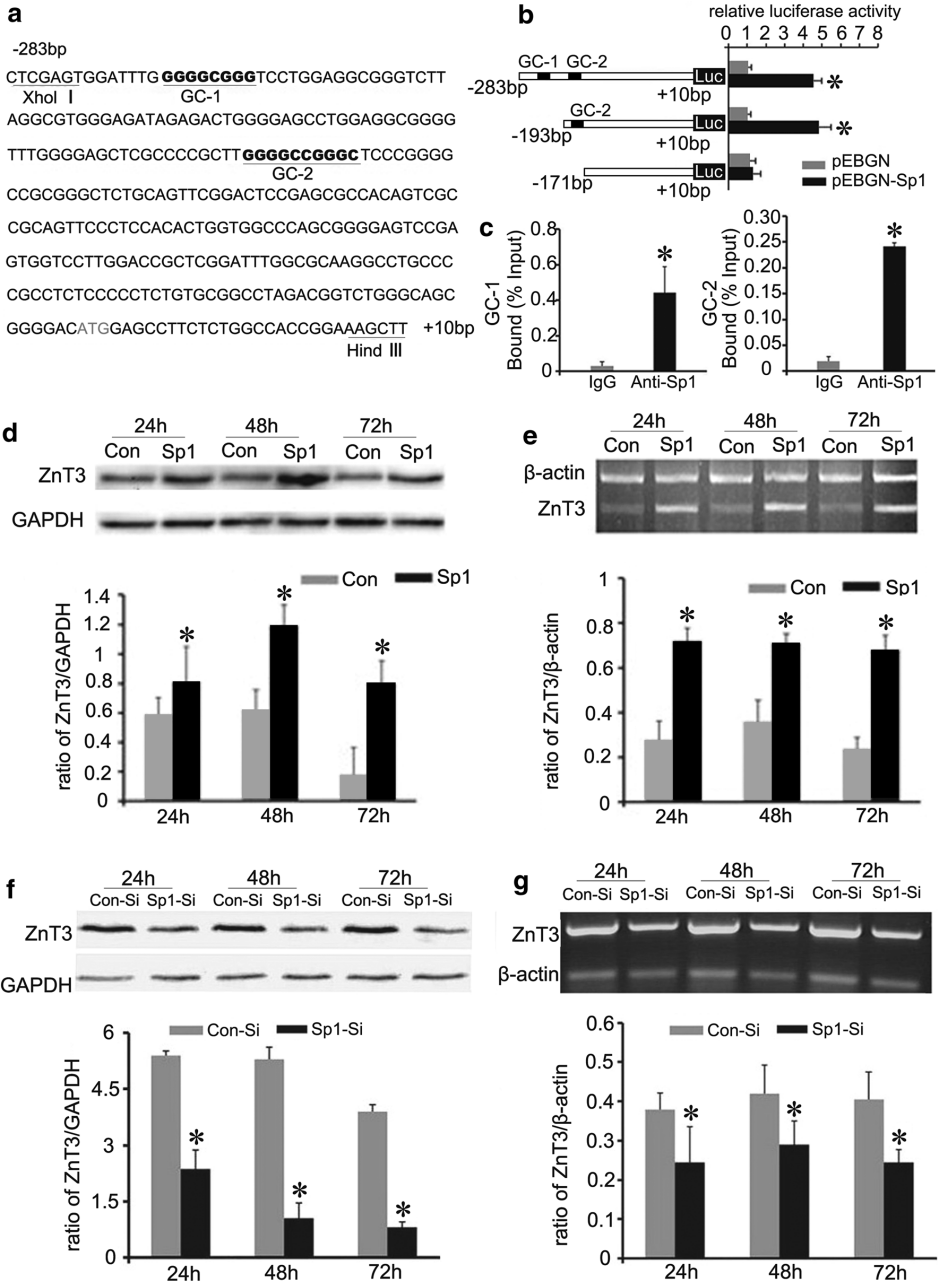
Sp1 up-regulates ZnT3 gene transcriptional activity. **a** The scheme chart of the mouse ZnT3 promoter sequence. Potential Sp1 binding sites (GC-1 and GC-2) are underlined. **b** Dual-luciferase (Luc) report gene assay of the targeting relationship of Sp1 and ZnT3. Sp1 activates ZnT3 transcription by binding to GC boxes in ZnT3 promoter. n = 3 *p < 0.05 compared to BHK cells transfected with empty pEBGN vector. (C) ChIP analysis of Sp1 binding to ZnT3 promoter. The relative amounts of ZnT3 promoter were normalized to input DNA. GC-1: ZnT3 promoter (−338 to  −232); GC-2:ZnT3 promoter (−253 to  −112). n = 3. *p < 0.05 compared to control IgG. **d–g** The effect of Sp1 on ZnT3 expression. ZnT3 expression level greatly increase in BHK cells overexpressing Sp1 (**d** Western blot analysis, **e** RT-PCR analysis). In contrast, ZnT3 expression level decreases in BHK cells transfected with Sp1 siRNA (**f** Western blot analysis, **g** RT-PCR analysis). n = 4. *p < 0.05 compared to BHK cells transfected with empty vector

Gene transcriptional dysregulation is greatly involved in the mechanisms of neurodegeneration in HD [35]. To clarify if the down-regulation of ZnT3 expression might result from transcriptional inhibition in HD, we assessed ZnT3 promoter via bioinformatics. The data hints that the promoter of mouse ZnT3 gene contains two GC-rich boxes, which might be Sp1 binding sites (Fig. 5a). Sp1, a key transcription factor isolated from human being, has been shown to recognize related GC motifs within promoter regions [36]. One GC-rich box (GC-1) in ZnT3 promoter is from −269 bp to −261 bp, the other (GC-2) is from −184 bp to −174 bp (Fig. 5a). These putative Sp1 sites are conserved among human, rat, and mouse. To verify whether Sp1 is a transcription factor of ZnT3, we first examined the effect of Sp1 on the ZnT3 promoter activity by using dual-luciferase reporter gene assay. The reporter activities of pGL3-ZnT3 (−283–+ 10) containing both GC-1 and GC-2 sequence and pGL3-ZnT3 (−193–+ 10) containing GC-2 sequence were significantly up-regulated in Sp1 transfected cells (Fig. 5b). However, the reporter activities of pGL3-ZnT3 (−171–+ 10) without putative Sp1 binding sites displayed no differences in Sp1 transfected cells and control cells (Fig. 5b). This finding suggests that Sp1 affects the promoter activity of ZnT3 through GC boxes.

To further demonstrate if Sp1 transactivates ZnT3 by binding GC boxes in the promoter, we pursued chromatin immunoprecipitation (ChIP) assay. Sp1 antibody precipitated more DNAs containing these ZnT3 promoter sequences with GC-1 or GC-2 than a mock immunoprecipitation with control IgG (Fig. 5c). Additionally, Sp1 overexpression significantly promoted the expression of endogenous ZnT3 protein and mRNA in WT BHK cells (Fig. 5d, e and Additional file 1: Figure S2). In contrast, knockdown of Sp1 resulted in a decline in ZnT3 protein and mRNA levels (Fig. 5f, g and Additional file 1: Figure S3). Consequently, these results imply that Sp1 plays a key role in transcriptional regulation of ZnT3 gene.

### Mutant huntingtin disturbs ZnT3 transcription by inhibiting the binding of Sp1 to ZnT3 promoter

**Fig. 6:**
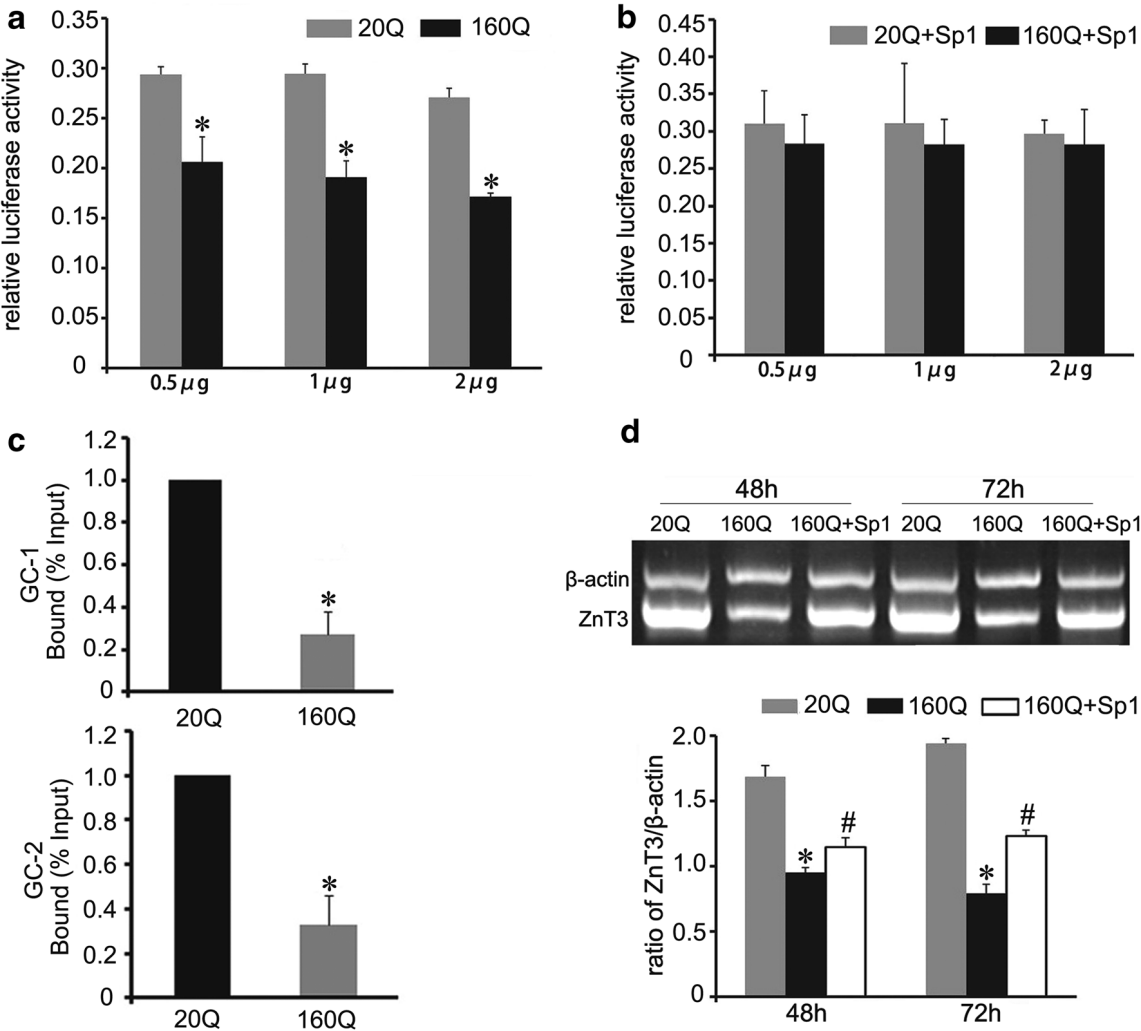
Mutant huntingtin inhibits the binding of Sp1 to ZnT3 promoter. **a** Dual-luciferase report gene assay shows that the promoter activity of ZnT3 is down-regulated in BHK cells transfected with different concentrations of 160Q Htt vector. n = 3. *p < 0.05 compared to 20Q cells. **b** Dual-luciferase report gene assay shows that Sp1 overexpression up-regulates ZnT3 promoter transcriptional activity in 160Q BHK cells. n = 3. **c** ChIP analysis shows that mHtt inhibits the binding of Sp1 to ZnT3 promoter containing GC-1 box or GC-2 box. The relative amounts of ZnT3 were normalized to input DNA. n = 3. *p < 0.05 compared to 20Q Htt. **d** RT-PCR analysis shows that forced overexpressed Sp1 reverses the suppression of ZnT3 mRNA by mHtt in 160Q BHK cells. n = 3, *p < 0.05 compared to 20Q cells. #*p *< 0.05 compared to 160Q cells

We further investigated the effect of mHtt on ZnT3 transcription activity. Different concentrations of mHtt vector (0.5 µg, 1 µg, 2 µg) were respectively co-transfected with reporter plasmid pGL3-ZnT3 (−283–+ 10) into BHK cells. Dual-luciferase reporter gene assay showed that 160Q Htt reduced the ZnT3 promoter activity, compared to 20Q Htt (Fig. 6a).

Mutant huntingtin has been demonstrated to inhibit the binding of Sp1 to target genes in HD [35, 36]. In this report, ChIP assay displayed that there was a significant decrease in Sp1-immunoprecipitated DNA consisting of ZnT3 promoter in 160Q cells, compared to 20Q cells (Fig. 6c). However, overexpression of Sp1 ameliorated ZnT3 promoter activity (Fig. 6b) and up-regulated ZnT3 mRNA level in 160Q cells (Fig. 6d). In summary, these data indicate that mHtt disturbs ZnT3 transcription by inhibiting the binding of Sp1 to ZnT3 promoter, and Sp1 reverses the suppression of mHtt to ZnT3 transcription activity.

## Discussion

Impairment of synaptic function contributes to the HD pathogenesis. Vesicular zinc is of significance for synaptic function. Herein, we identified a significant loss of total zinc, especially vesicular zinc in the brain of the HD transgenic mice. Furthermore, a reduction of ZnT3 expression was observed in these mice and 160Q BHK cells. ZnT3, an important protein located on the membrane of synaptic vesicles, affects synaptic function via various mechanisms in neurons [28]. Most important of all, ZnT3 is responsible for the movement of zinc from the cytoplasm to the synaptic vesicles. Vesicular zinc level is dependent on ZnT3 protein abundance. Zinc is eliminated from synaptic vesicles in the brain of ZnT3 knockout mice [27]. In the present study, we found that vesicular zinc detected by AMG staining is dramatically decreased in the striatum, hippocampus and cortex of TG mice compared to age-matched controls, suggesting that the reduced ZnT3 expression in HD greatly disturbs zinc homeostasis in synaptic vesicles.

It is well-known that brain zinc homeostasis is strictly controlled to guarantee physiological function under healthy conditions. Vesicular zinc serves as a signal factor in a subclass of glutamatergic neurons, which is linked to glutamate signaling and cognitive activity [37, 38]. Specially, vesicular zinc can inhibit glutamate release. In mutant mice lacking vesicular zinc, glutamate release inhibition induced by zinc is absent [39]. Several lines of evidence support a key role of glutamate-mediated excitotoxic cell death in HD pathogenesis [40, 41]. Thus, it appears likely that loss of vesicular zinc in TG mice aggravates glutamate-mediated excitotoxic neuron death. On the other side, vesicular zinc signaling meets the requirement for cognitive and emotional behavior [27]. Movement impairment and cognitive deficits occur prior to neuron degeneration in HD patients [6]. Our results reveal that vesicular zinc level is lower in the hippocampus of TG mice compared to WT mice. Consequently, the decrease of vesicular zinc might be responsible for the cognitive decline in HD.

Beside affecting vesicular zinc, the reduced ZnT3 expression may also impair synaptic structures and proteins. In ZnT3 knockout mice, there is a decrease in total dendritic spines per neuron. Similarly, synaptic plasticity-related proteins, such as presynaptic synaptosome-associated protein 25 (SNAP25) and postsynaptic PSD95, are markedly decreased in the absence of ZnT3 [27]. It has been reported that both SNAP25 and PSD95 are also defective in HD [42, 43]. Of note is that TG mice showed significant reductions in ZnT3 protein and mRNA levels at a relatively early stage of this disease (about 14 weeks). Subtle alterations in synaptic function can lead to the early symptoms of HD [44]. In consequence, we conclude that reduction of ZnT3 expression in the HD brain may result in synaptic dysfunction.

One of distinctive characteristics of mHtt is to form aggregates or inclusions, which directly recruit synaptic proteins [45, 46]. To understand how mHtt causes the reduction of ZnT3 expression in HD, subsequently, we first examined whether mHtt aggregates could recruit ZnT3 protein. Co-immunoprecipitation results showed that ZnT3 protein was not detected in aggregates, suggesting that down-regulation of ZnT3 does not come from the recruit of mHtt into aggregates. Additionally, mHtt affects gene expression via altering the activity of transcription factors or via abnormally interacting with them [35, 47, 48]. Two major transcriptional pathways, CRE and Sp1-mediated transcription, have been extensively studied in HD [35]. Increasing findings demonstrate that several genes containing Sp1-binding motifs in their promoters are down-regulated in HD [47, 48]. In the present study, one important finding was that Sp1 transactivated ZnT3 gene by binding the GC boxes in the promoter. We also detected whether mHtt could reduce ZnT3 mRNA level through affecting Sp1 expression. Interestingly, TG mice displayed an increasing Sp1 expression compared to WT mice, which was consistent with previous reports [49]. This result indicates that inhibition of ZnT3 expression does not result from decreased Sp1 protein.

More importantly, Sp1 is found to interact with Htt [36, 50, 51]. mHtt binds to C-terminal region of Sp1, which inhibits the interaction of Sp1 with its targeting gene promoters [36, 51]. Here, we confirmed that mHtt inhibits the binding of Sp1 to the GC boxes in the ZnT3 gene promoter so as to reduce ZnT3 expression. Similar mechanism has been verified in previous studies. For example, Sp1 can bind to the GC boxes in the nerve growth factor receptor (NGFR) gene promoter, followed by activating the expression of NGFR. mHtt inhibits the binding of Sp1 to the NGFR promoter to decrease its transcription [36]. As mentioned above, TG mice showed the defective ZnT3 expression at a relatively early stage of this disease. Given that Sp1 disruption also occurs early in human HD pathogenesis, even in postmortem tissues of pre-symptomatic grade [36, 47]. We reasonably infer that mHtt blocks the binding of Sp1 to ZnT3 gene promoter to decrease the expression of ZnT3, thereby disrupting vesicular zinc homeostasis. In order to further confirm this mechanism, we investigated the influence of overexpression of Sp1 on ZnT3 mRNA level in 160Q BHK cells. As expected, overexpression of Sp1 enhanced the transcriptional activity of ZnT3 to up-regulate its mRNA level in our experiments. Thus, overexpression of Sp1 greatly attenuates inhibition of the binding of Sp1 to ZnT3 promoter by mHtt. Obviously, increasing endogenous Sp1 protein in TG mice might be a reactive result as reduction of binding between Sp1 and ZnT3 promoter.

## Conclusions

**Fig. 7:**
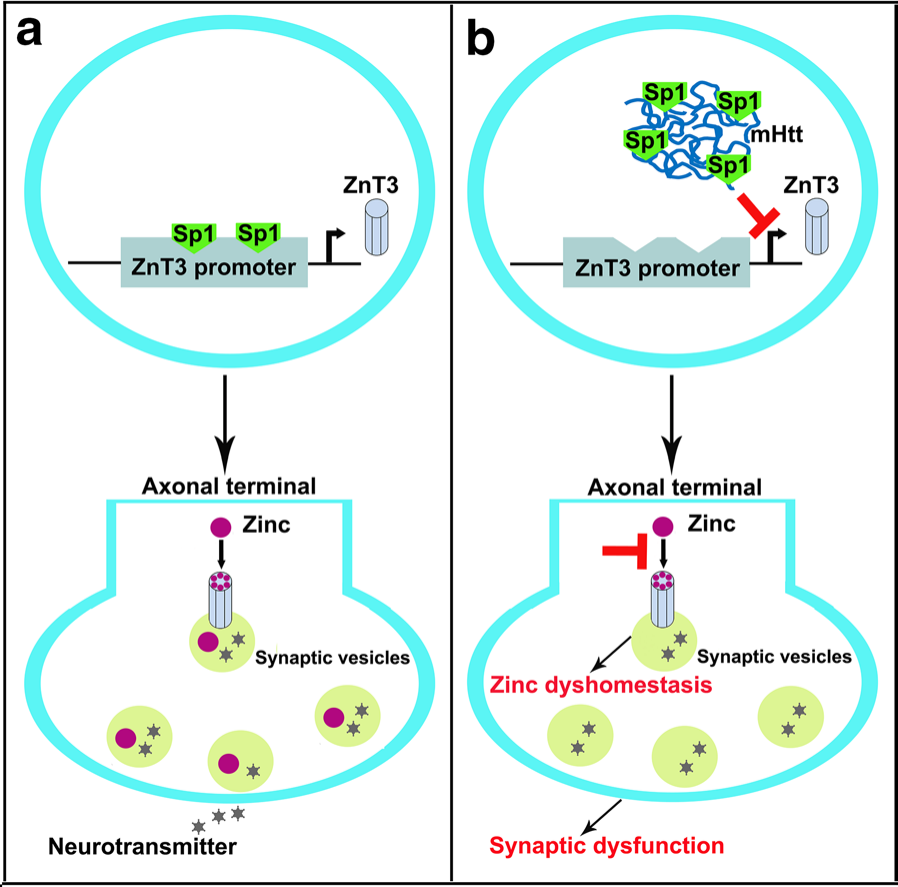
The mechanism of ZnT3 down-regulation and its effect on vesicular zinc in HD. **a** Transcription factor Sp1 binds to ZnT3 promoter and up-regulates its expression. Vesicular zinc is dependent on ZnT3 on the membrane of synaptic vesicles in the axonal terminal. **b** mHtt inhibits the binding of Sp1 to ZnT3 promoter. Down-regulated ZnT3 expression reduces the transport of zinc into synaptic vesicles. Zinc dyshomestasis contributes to synaptic dysfunction

This work has identified significant reductions in vesicular zinc and the underlying molecular mechanism in HD (Fig. 7). ZnT3 expression is decreased in the brain at the early stage of TG mice, indicating that altered neuronal zinc homestasis is an early event in HD pathogenesis. Sp1 serves as one important transcription factor of ZnT3 to activate its transcription. However, mHtt down-regulates ZnT3 expression by inhibiting the binding of Sp1 to the GC boxes in ZnT3 promoter, which further leading to a decrease in the transport of zinc into synaptic vesicles from cytoplasm. The decline in vesiclular zinc induced by mHtt results in synapse dysfunction and cognitive impairment. Our findings provide novel insights to elucidate the pathogenesis of HD and pave new therapeutic avenues for the treatment of HD.

## Methods

### Huntington’s disease transgenic mice

B6C3-Tg (HD82Gln) 81Dbo/J (N171-HD82Q) HD transgenic mice (Jackson Laboratories) express a cDNA encoding a 171 amino acid N-terminal fragment of huntingtin containing 82 CAG (Q) repeats [34]. At the age of 4 weeks, mice were genotyped by polymerase chain reaction (PCR) of tail DNA genotyped according to the Jackson Laboratories protocol to determine hemizygosity for the HD transgene. Wild-type littermates were used as controls. All procedures for animal use were approved by the institutional Animal Care and Committee of Tongji Medical College, Huazhong University of Science and Technology.

### Plasmids

For the generation of ZnT3 promoter reporter constructs, pGL3-ZnT3 (−283–+ 10) vector containing both GC-1 and GC-2 boxes, pGL3-ZnT3 (−193–+ 10) vector containing GC-2 box and pGL3-ZnT3 (−171–+ 10) vector without any GC-rich element, the promoter fragments of wild type mice ZnT3 gene (GenBank accession number 22784) were amplified using the PCR technique and subcloned into pGL-3 basic vector harboring a luciferase reporter gene (Promega). The pEGFP-exon-1 20Q-Htt and pEGFP-exon-1 160Q-Htt plasmids, pEBGN-Sp1 plasmid, nuclear factor κB (NF-κB) p50 and NF-κB (p65) vectors, Wilms’ tumor (WT1) gene vectors (WT1 + KTS, WT1-KTS) and pMIC-Sp1 were kindly provided by Xiao-Jiang Li (USA), Gerald Thiel (Germany), Neil D. Perkins (UK), Holger Scholz (Germany), and Jacqueline Marvel (France), respectively.

### Cell culture and transfection

Baby hamster kidney cells (BHK cells), a specific cell line originally derived from kidney tissue of baby hamster, were cultured as previously described [34]. The cells were planted in the six-well plate. After 24 h, they were transiently transfected with plasmids using Lipofectamine TM 2000 (Invitrogen). The cells were replaced with fresh culture medium 6 h after transfection.

### Flame atomic absorption spectrometry (FAAS)

The 20-week-old N171-82Q mice and age-matched WT mice (n = 3) were sacrificed after anesthesia with pentobarbital sodium intraperitoneal injection (40–45 mg/kg body weight). After the brain tissues were dissected and weighed, samples were washed and digested in ultrapure nitric acid. All the samples were evaporated to dryness, and were resuspended in 2% nitric acid. After the resuspending of the samples, the total zinc was detectable by SpectrAA-240FS (VaRIAN).

### Autometallography (AMG)

The 20-week-old N171-82Q mice and age-matched WT mice (n = 3) were anesthetized and perfused transcardially with 4% paraformaldehyde containing 0.25% glutaraldehyde after being perfused with 0.3% sodium sulfide solution.

For light microscopy (LM), the samples were cut into 10 μm sections in a cryostat and mounted on glass slides. The sections were placed in Farmer-cleaned jars and immersed in the AMG developer [52]. The jars were placed in a water bath at 26 °C and shook for 1 h. Then the AMG development was stopped with a 5% sodium thiosulfate solution for 10 min. The sections were rinsed with water at 38 °C for 10 min. Images were taken on a Nikon microscope (Digital Camera DXM 1200).

For electron microscopy (EM), the samples after AMG development were fixed with 1% osmium acid and embedded in epon. Then, the samples were cut into 60 nm ultrathin sections and were stained with uranyl citrate and lead acetate. Finally, the sections were examined with a transmission electron microscope (FEI Tecnai G^2^ Type 12).

### Immunohistochemistry

Immunohistochemical staining of tissues sections was performed as previously described [34]. Briefly, the 20-week-old N171-82Q mice and age-matched WT mice (n = 3) were deeply anesthetized and perfused transcardially with saline, followed by 4% paraformaldehyde in 0.1 M PB 150 ml. The sections were incubated with polyclonal rabbit anti-ZnT3 antibodies (1:100, Proteintech) overnight at 4 °C followed by biotinylated anti-rabbit IgG (1:200, Vector Labs) for 2 h and a vidinbiotin complex (1:100, Vector ABC Elite; Vector Labs) for 1 h at room temperature. Then, the sections were incubated 0.02% diaminobenzidine (Sigma-Aldrich) and 0.005% hydogen peroxide in 0.05 M Tris–HCl buffer for 10 to 15 min. Images were taken on a Nikon microscope (Digital Camera DXM 1200).

### Western blot analysis

Brain tissues from 14-, 18-, and 20-week-old N171-82Q mice and age-matched WT mice (n = 4 at each age) and BHK cells at different time after transfection (n = 4 at each time interval) were lysed in RIPA buffer supplemented with protease and phosphatase inhibitors (Sigma-Aldrich). Protein samples were resolved by SDS-PAGE and transferred onto NC membranes. Blots were incubated in primary antibody (1:1000 for ZnT3, 1:1000 for Sp1, 1:10,000 for γ-tubulin and 1:5000 for GAPDH) overnight at 4 °C followed by horseradish peroxidase-conjugated secondary antibody for 2 h at room temperature. The immunoreactive bands were visualized by exposure to an enhanced chemiluminescence (ECL) kit (Pierce Thermo Scientific).

### Rt-pcr

Total RNA from BHK cells, wild type and HD transgenic mice was extracted with Trizol reagent (Invitrogen). ZnT3 and Sp1 mRNA was amplified by reverse-transcription polymerase chain reaction, and β-actin mRNA was taken as an internal control. The following PCR conditions were used: 94 °C for 3 min; 30 cycles of denaturing at 94 °C for 30 s, annealing for 30 s, extension at 72 °C for 45 s, and a final extension at 72 °C for 3 min. Photos of the amplified genes were taken after agarose gel (1%) electrophoresis. The primers and amplification used in the PCRs were listed in the Table 1.

**Table 1:** Primers, amplicon size and annealing temperature of ZnT3, Sp1 and β-actin for RT-PCR and ChIP-PCR

Name	Primer sequences		Amplicon size (bp)	Annealing temperature
	Forward	Reverse		
β-actin	GTCGTACCACAGGCATTGTGATGG	GCAATGCCTGGGTACATGGTGG	492	58/63 °C
β-actin	TTTCCAGCCTTCCTTCTTGGGTATG	ATAGAGGTCTTTACGGATGTCAACG	100	58/63 °C
ZnT3	GGAGGTGGTTGGTGGGTATTTAGC	CCATTAGCAGATTGGCACAGACAGC	317	58 °C
Sp1	AGAACCCACAAGCCCAGACAATC	CTCCTTCTCCACCTGCTGTCTCA	330	63 °C
ZnT3 promoter -338 ~ -232(GC-1)	GTCGTACCACAGGCATTGTGATGG	GCAATGCCTGGGTACATGGTGG	106	60 °C
ZnT3 promoter -253 ~ -112(GC-2)	TTTCCAGCCTTCCTTCTTGGGTATG	ATAGAGGTCTTTACGGATGTCAACG	141	60 °C

### siRNA transfection

The sense Sp1 siRNA (5′-GGAUGGUUCUGGUCAAAUA-3′) and control non-silencing siRNA were produced by RIBOBIO company (China). The BHK cells were grown in Opti-MEM till 60% confluent. The cells were transfected with siRNA (50 nM) using Lipofectamine TM 2000 (Invitrogen). The cells were harvested at 48 h after transfection. RT-PCR and Western blot assay were respectively performed to dectect protein and mRNA levels of Sp1.

### Dual-luciferase reporter gene assay

Dual-luciferase reporter gene assays were performed according to the manufacturer’s protocol of dual-luciferase reporter assay system (Promega). For the detection of targeting relationship of Sp1 and ZnT3, pEBGN-Sp1 was co-transfected with reporter plasmids pGL3-ZnT3 (−283–+ 10), pGL3-ZnT3 (−193–+ 10), or pGL3-ZnT3 (−171–+ 10), respectively, into BHK cells. pEBGH empty vector was served as a control. For examining the effect of mHtt on ZnT3 transcription activity, different concentration of mHtt vector (0.5 µg, 1 µg, 2 µg) was co-transfected with reporter plasmid pGL3-ZnT3 (−283–+ 10) into BHK cells. 20Q Htt vector was served as a control. Cells were harvested at 48 h after transfection. Relative luminescence units (RLUs) produced by firefly luciferase activity were measured by using LB9507 luminometer. Luciferase activity of pRL-TK Renilla luciferase plasmid was used to determine the background for each luciferase reaction. Luciferase activity = RLU (firefly luciferase)/RLU (Renilla luciferase). All experiments were performed three times, and the mean relative luciferase activity was obtained.

### Chromatin immunoprecipitation assay (ChIP)

ChIP assays were performed using the Magna ChIP A Kit (Millipore) according to the manufacturer’s protocol. BHK cells were seeded in 10 cm dishes. 10 µg of pre-immune rabbit IgG or anti-Sp1 antibody (ChIP Grade, Abcam) was used for each ChIP reaction. Precipitated DNA was analyzed using a 7500 Real-Time PCR System (Applied Biosystems). The ZnT3 promoter primers used for ChIP-PCR assay are included in Table 1. The amount of amplified DNA was expressed as percent of the input. All ChIP assays were performed three times.

### Statistical analysis

Statistical analyses were carried out using SPSS 17.0 software for one-way ANVOA followed by Student’s *t* test. All values were represented as mean ± S.D. Differences were considered significant if *p *< 0.05.

## Supplementary information

**Additional file 1: Figure S1.** Total zinc level in TG mice brain. Total zinc decreases in the cortex, striatum and hippocampus of the 20-week-old TG mice compared to age-matched WT mice. n = 3. *p < 0.05 compared to WT mice. **Figure S2.** Effect of transcription factors overexpression on ZnT3 expression in WT BHK cells. Overexpression of Sp1 increases ZnT3 protein levels. However, overexpression of other transcription factors, WT1 and NF-κB, does not affect ZnT3 expression. **Figure S3.** Sp1 expression level in WT BHK cells transfected with Sp1 siRNA. Sp1 siRNA effectively knocks down Sp1 expression in WT BHK cells. **a, b** are Western blots analysis of ZnT3 expression and RT-PCR analysis of ZnT3 mRNA level in BHK cells transfected with different concentrations (0, 5, 25, 50 μg) of Sp1 siRNA vectors, respectively. Con-Si: control siRNA; Sp1-Si: Sp1 siRNA

## Data Availability

All data generated or analysed during this study are included in this published article and its Additional files.

## Abbreviations

HD: Huntington’s disease
ZnT3: zinc transporter 3
Htt: Huntingtin
mHtt: Mutant huntingtin
BHK cells: Baby hamster kidney cells
FAAS: Flame atomic absorption spectrometry
AMG: Autometallography
ChIP: Chromatin immunoprecipitation assay
