# Supplementary material for: Disruption of zinc transporter ZnT3 transcriptional activity and synaptic vesicular zinc in the brain of Huntington’s disease transgenic mouse
Source: Cell Biosci. 2020 Sep 11;10:106. doi: 10.1186/s13578-020-00459-3 (PMC7488477; doi:10.1186/s13578-020-00459-3)
Supplement: Supplementary file 1 — Additional file 1: Figure S1. Total zinc level in TG mice brain. Total zinc decreases in the cortex, striatum and hippocampus of the 20-week-old TG mice compared to age-matched WT mice. n = 3. *p < 0.05 compared to WT mice. Figure S2. Effect of transcription factors overexpression on ZnT3 expression in WT BHK cells. Overexpression of Sp1 increases ZnT3 protein levels. However, overexpression of other transcription factors, WT1 and NF-κB, does not affect ZnT3 expression. Figure S3. Sp1 expression level in WT BHK cells transfected with Sp1 siRNA. Sp1 siRNA effectively knocks down Sp1 expression in WT BHK cells. a, b are Western blots analysis of ZnT3 expression and RT-PCR analysis of ZnT3 mRNA level in BHK cells transfected with different concentrations (0, 5, 25, 50 μg) of Sp1 siRNA vectors, respectively. Con-Si: control siRNA; Sp1-Si: Sp1 siRNA [file 13578_2020_459_MOESM1_ESM.doc]

***Supplementary material***

**Disruption of zinc transporter ZnT3 transcriptional activity and synaptic vesicular zinc in the brain of Huntington's disease transgenic mouse**

**Li Niu^1^, Li Li^2^, Shiming Yang^1^, Weixi Wang^1^, Cuifang Ye^1*^, He Li^1,3*^**

^1^Department of Histology and Embryology, Tongji Medical College, Huazhong University of Science and Technology, Wuhan 430030, P.R. of China

^2^School of Biomedical Sciences, LKS Faculty of Medicine, the University of Hong Kong, Hong Kong S.A.R., P. R. of China

^3^Hubei Key Laboratory of Embryonic Stem Cell Research, Hubei University of Medicine, Shiyan 442000, P. R. of China

**Li Niu**, Department of Histology and Embryology, Tongji Medical College, Huazhong University of Science and Technology, 13 # Hangkong Road, Wuhan 430030, P. R. of China. Phone: 86-27-8369-2612; Fax: 86-27-8369-2718; E-mail: [2794907@qq.com](mailto:2794907@qq.com)

**Li Li,** School of Biomedical Sciences, LKS Faculty of Medicine, the University of Hong Kong, Hong Kong S.A.R., P. R. of China; E-mail: mrcatking@gmail.com

**Shiming Yang**, Department of Histology and Embryology, Tongji Medical College, Huazhong University of Science and Technology, 13 # Hangkong Road, Wuhan 430030, P. R. of China. Phone: 86-27-8369-2612; Fax: 86-27-8369-2718; E-mail: 858382929@qq.com

**Weixi Wang**, Department of Histology and Embryology, Tongji Medical College, Huazhong University of Science and Technology, 13 # Hangkong Road, Wuhan 430030, P. R. of China. Phone: 86-27-8369-2612; Fax: 86-27-8369-2718; E-mail: 834115090@qq.com

**Cuifang Ye**, Department of Histology and Embryology, Tongji Medical College, Huazhong University of Science and Technology, 13 # Hangkong Road, Wuhan 430030, P. R. of China. Phone: 86-27-8369-2612; Fax: 86-27-8369-2718; E-mail: [yecf@tjmu.edu.cn](mailto:yecf@tjmu.edu.cn)

**He Li,** Department of Histology and Embryology, Tongji Medical College, Huazhong University of Science and Technology, 13 # Hangkong Road, Wuhan 430030, P. R. of China. Phone: 86-27-8369-2612; Fax: 86-27-8369-2718; E-mail: [heli@tjmu.edu.cn](mailto:heli@tjmu.edu.cn). Department of Histology and Embryology, Hubei University of Medicine, Shiyan 442000, P. R. of China

**^*^ Correspondence: Dr. Cuifang Ye (**[**yecf@tjmu.edu.cn**](mailto:yecf@tjmu.edu.cn)**)**

**Prof. He Li (**[**heli@tjmu.edu.cn**](mailto:heli@tjmu.edu.cn)**)**

**Contents**

Fig. S1 6

Fig. S2 7

Fig. S3 8


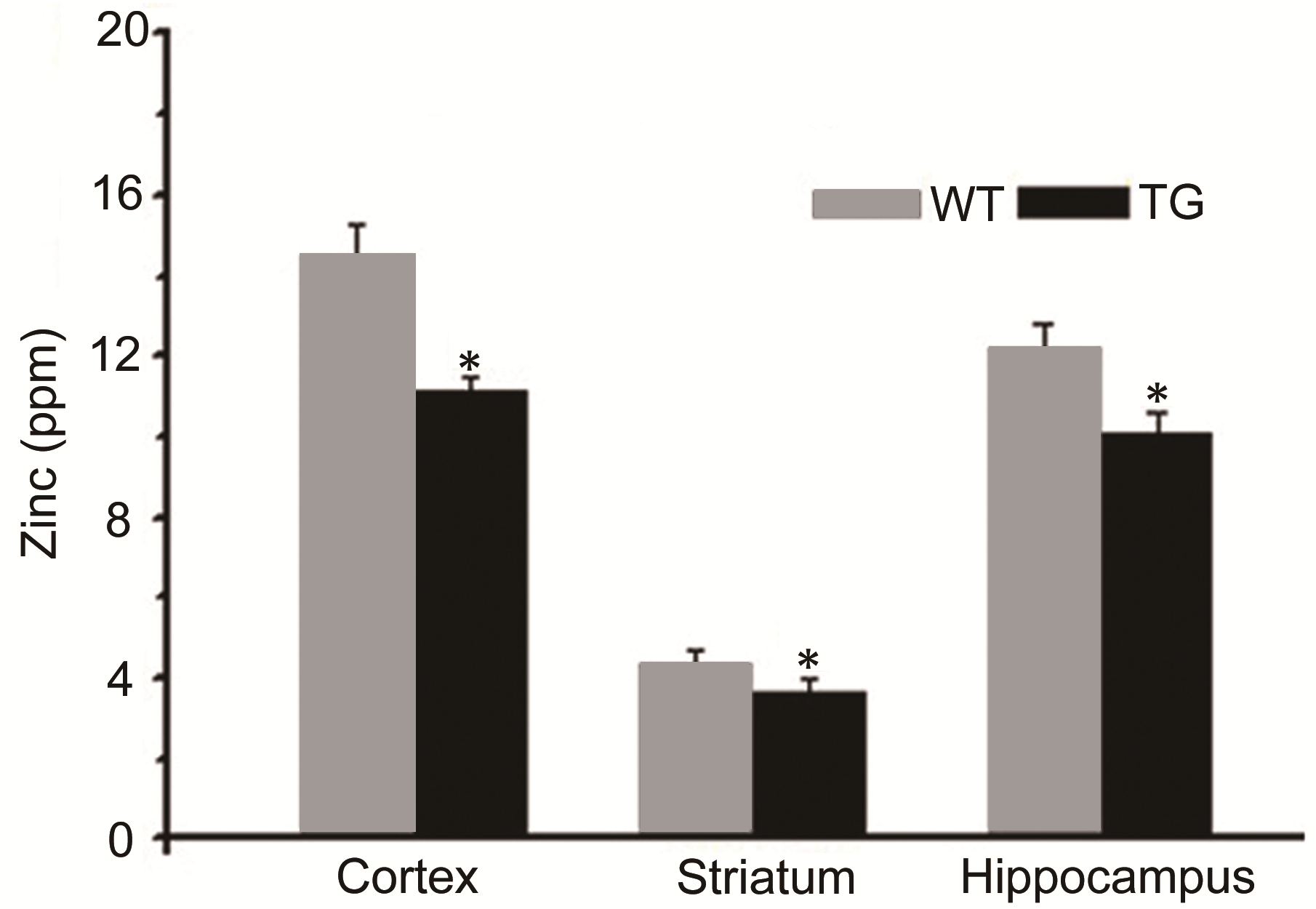


**Fig. S1** Total zinc level in TG mice brain. Total zinc decreases in the cortex, striatum and hippocampus of the 20-week-old TG mice compared to age-matched WT mice. n = 3. *, *p* < 0.05 compared to WT mice.


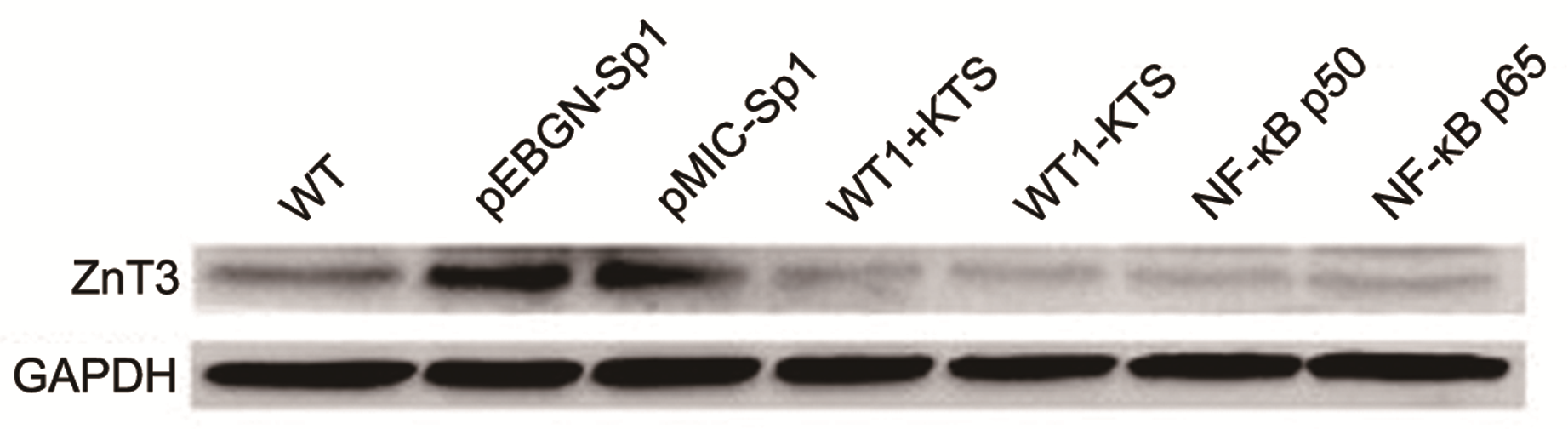


**Fig. S2** Effect of transcription factors overexpression on ZnT3 expression in WT BHK cells. Overexpression of Sp1 increases ZnT3 protein levels. However, overexpression of other transcription factors, WT1 and NF-κB, does not affect ZnT3 expression.


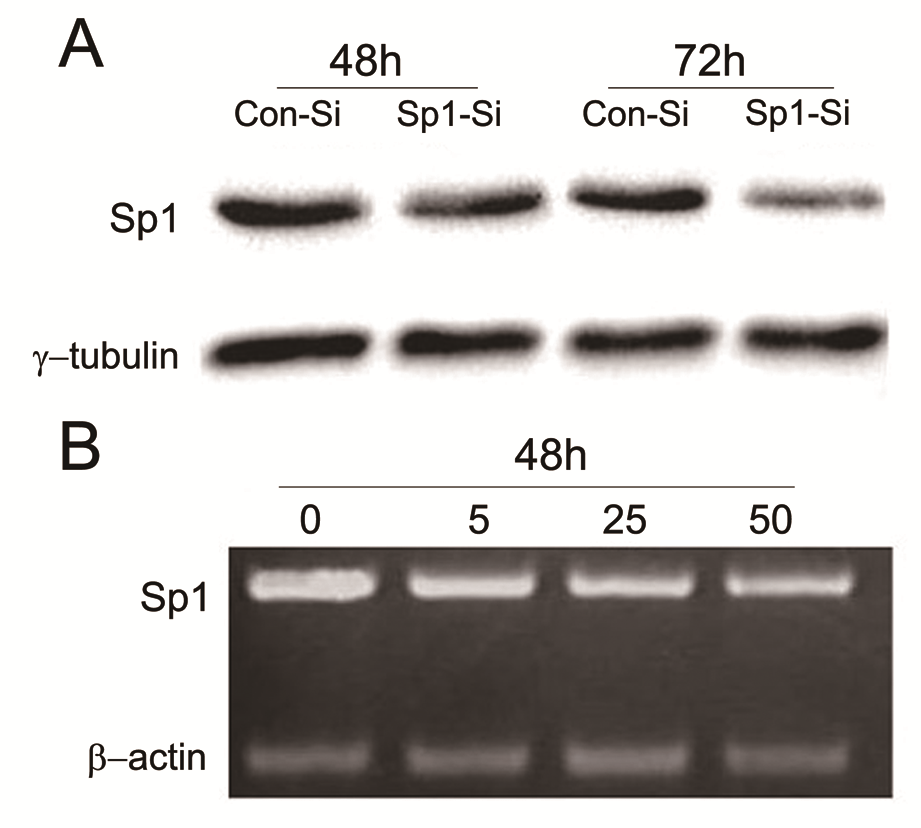


**Fig. S3** Sp1 expression level in WT BHK cells transfected with Sp1 siRNA. Sp1 siRNA effectively knocks down Sp1 expression in WT BHK cells. A and B are Western blots analysis of ZnT3 expression and RT-PCR analysis of ZnT3 mRNA level in BHK cells transfected with different concentrations (0, 5, 25, 50μg) of Sp1 siRNA vectors, respectively. Con-Si: control siRNA; Sp1-Si: Sp1 siRNA.
